# Metabolic flexibility during sleep

**DOI:** 10.1038/s41598-021-97301-8

**Published:** 2021-09-08

**Authors:** Simeng Zhang, Yoshiaki Tanaka, Asuka Ishihara, Akiko Uchizawa, Insung Park, Kaito Iwayama, Hitomi Ogata, Katsuhiko Yajima, Naomi Omi, Makoto Satoh, Masashi Yanagisawa, Hiroyuki Sagayama, Kumpei Tokuyama

**Affiliations:** 1 International Institute for Integrative Sleep Medicine (WPI-IIIS), University of Tsukuba, 1-1-1 Tennodai, Tsukuba, Ibaraki Japan; 2 Doctoral Program in Sports Medicine, Graduate School of Comprehensive Human Sciences, University of Tsukuba, Ibaraki, Japan; 3 Ph.D. Program in Human Biology, Doctoral Program in School of Integrative and Global Majors (SIGMA), University of Tsukuba, Ibaraki, Japan; 4 Faculty of Budo and Sport Studies, Tenri University, Nara, Japan; 5 Graduate School of Integrated Arts and Sciences, Hiroshima University, Hiroshima, Japan; 6 Faculty of Pharmaceutical Sciences, Josai University, Saitama, Japan; 7 Faculty of Health and Sports Sciences, University of Tsukuba, Tsukuba, Ibaraki Japan

**Keywords:** Metabolism, Homeostasis, Metabolic diseases

## Abstract

Known as metabolic flexibility, oxidized substrate is selected in response to changes in the nutritional state. Sleep imposes an extended duration of fasting, and oxidized substrates during sleep were assumed to progressively shift from carbohydrate to fat, thereby gradually decreasing the respiratory quotient (RQ). Contrary to this assumption, whole-room indirect calorimetry with improved time resolution revealed that RQ re-ascended prior to awakening, and nadir of RQ in non-obese young adults occurred earlier in women than men after bedtime. The transient decrease in RQ during sleep was blunted in metabolically inflexible men with smaller amplitude of diurnal rhythm in RQ. Similarly, the effect of 10 years difference in age on RQ became significant during sleep; the decrease in RQ during sleep was blunted in older subjects. Inter-individual difference in RQ become apparent during sleep, and it might serve as a window to gain insight into the early-stage pathogenesis of metabolic inflexibility.

## Introduction

Metabolic flexibility, defined by Kelley et al., is the ability of leg muscles to switch from predominantly fat oxidation to carbohydrate oxidation under insulin-stimulated conditions after overnight fasting^1–3^. The scope of metabolic flexibility has since been extended to encompass the response to cyclic changes in the nutritional state at the whole-body level, which includes acute responses of energy metabolism to meals and sleep^4–6^. Smaller amplitude of the 24-h RQ rhythm due to elevated nocturnal values is an early-stage phenotype of metabolically inflexible individuals^6^. Disrupted metabolic flexibility, or metabolic inflexibility, is associated with pathological conditions including obesity, metabolic syndrome and type 2 diabetes mellitus^5,7^.

Most people are monophasic sleepers, getting all of their rest in one long sleep episode, and the extended duration of fasting imposes a metabolic consequence. It has been assumed that RQ progressively decreases during sleep^4^. However, an improved time resolution of the whole-room indirect calorimetry revealed a seemingly counterintuitive finding that RQ reaches its nadir, and then re-ascends after midnight^8,9^. The U-shaped time course of the RQ during sleep suggests that sleeping energy metabolism does not simply reflect a transition of the nutritional state from postprandial to postabsorptive^8^.

Like the U-shaped time course of RQ during sleep, core body temperature declines and reaches its nadir after sleep onset, followed by a gradual increase before awakening. Daily rhythm of body temperature, a marker of the circadian pacemaker’s phase and amplitude, advances to an earlier hour and amplitude reduces in older people. The age-related shift and reduction of core body temperature amplitude is mainly due to advanced and elevated nocturnal values in the older people^10^. Difference in circadian phase of body temperature is also observed between men and women; women reach their nocturnal minimum body temperatures earlier than men^11–13^. In addition, both the beginning and end of the biologic night as defined by the onset and offset of melatonin secretion are earlier in women than in men^12^. Previous studies paid little attention to sex differences in sleeping energy metabolism; subjects were mainly men, and only occasionally included both men and women but the data was not analyzed by sex^6,8,14^. Given a similarity in time course of RQ and core body temperature during sleep, we hypothesized age- and sex-related differences in time course of energy metabolism during sleep. Sex differences may also exist in sleep quality and duration^15,16^, and it was invaluable to monitor sleep during indirect calorimetry.

In the present study, as an index of metabolic flexibility, we assessed the time course of the RQ during sleep by performing indirect calorimetry using a whole room metabolic chamber^17^. A U-shaped time course was examined by applying statistical rigor to our database of sleeping energy metabolism, which suggests that sleeping RQ might serve as a window to gain insight into the early-stage pathogenesis of metabolic inflexibility. The observed sex-related differences in the time course of RQ revealed possible mechanisms underlying the fuel selection during sleep.

## Methods

### Database

The present study was based on 127 recordings of indirect calorimetry in adults without obesity, which comprises sedentary or placebo control groups of our published studies addressing the effect of exercise^18–20^, skipping breakfast^21^ or subacute ingestion of oolong tea^9^ on 24-h energy metabolism, and the effect of exercise on peripheral clock gene expression^22^. Data from indirect calorimetry studies focusing on differences in energy metabolism among sleep stages^8^, effect of menstrual cycle on energy metabolism^23^, effect of exercise on sleep quality^24^, and effect of body pillow use on sleeping posture^25^ were also included in the present study. Sex differences in the RQ time course during sleep were not discussed in all of our previous studies, and the RQ time course over the 24 h was not presented in our previous studies except the recent study^9^. All of the 24 h indirect calorimetry presented in the present study was designed to achieve an individual energy balance^9,18–22^. All subjects were adults without obesity (BMI < 30 kg/m^2^), without current medical conditions, nonsmokers, and not taking any medications at the time of the study. Before the study began, the nature, purpose, and risks of the study were explained to all the subjects, and informed written consent was obtained. All study protocols were approved by the local ethics committee of the University of Tsukuba, and conducted in accordance with the Helsinki Declaration (Table 1).

**Table 1:** Approval ID by the local ethics committee, and Clinical Trials Registry of the studies.

Journal, year^reference^	Approval ID	Clinical Trials Registry, date
EBioMedicine, 2015^19^	Tai 24-44	Not registered
PLoS One, 2017^18^	Tai 24-119	Not registered
Metabolism, 2017^8^	Tai 22-390	Not registered
Am J Clin Nutr, 2019^21^	Tai 25-16	UMIN000032346, 2018/4/23
Nutrients, 2020^9^	H30-224	UMIN000035313, 2018/12/19
Metabolism Open, 2020^20^	Tai 26-46	UMIN000040638, 2020/6/3
J Appl Physiol, 2020 ^22^	Tai 28-32	UMIN000038252, 2019/10/10
Physiol Rep, 2020^23^	Tai 29-29	Not registered
Sci Rep, 2021^24^	Tai-28-52	UMIN000040428, 2020/5/31
Sleep Med Res, 2021^25^	H30-284	UMIN000035640, 2021/3/31

All study protocols were approved by the local ethics committee of the University of Tsukuba, and its approval ID was shown. Clinical Trials Registry was not applied for 4 observational studies^8,18,19,23^.

### Measurements

#### Indirect calorimetry

Energy metabolism was measured in a room-size metabolic chamber (Fuji Medical Science, Chiba, Japan). The airtight chamber measured 2.00 × 3.45 × 2.10 m, with an internal volume of 14.49 m^3^. The chamber was furnished with a bed, desk, chair, and toilet, and prescribed diet was provided through a “pass-through” box. The temperature and relative humidity of the incoming fresh air were controlled at 25.0 ± 0.5 °C and 55.0 ± 3.0%, respectively. Concentrations of oxygen (O_2_) and carbon dioxide (CO_2_) in the outgoing air were measured using an online process mass spectrometer (VG PrimaδB, Thermo Electron, Winsford, UK). The precision of the mass spectrometry, defined as the standard deviation of continuous measurement of the calibration gas mixture (O_2_ 15%–CO_2_ 5%), was demonstrated to be 0.0016% for O_2_ and 0.0011% for CO_2_, respectively. The O_2_ consumption (${\dot{\text{V}}}$O_2_) and CO_2_ production (${\dot{\text{V}}}$CO_2_) rates were calculated every 5 min using an algorithm providing improved transient response^17^. The RQ was calculated as the ratio of ${\dot{\text{V}}}$CO_2_/${\dot{\text{V}}}$O_2_, and energy expenditure was calculated from ${\dot{\text{V}}}$O_2_, ${\dot{\text{V}}}$CO_2_, and urinary nitrogen excretion ($\dot{\mathrm{N}}$)^26^. Non-protein RQ (NPRQ) was computed from the following equation: NPRQ = (${\dot{\text{V}}}$CO_2_ − 4.89 $\dot{\mathrm{N}}$)/(${\dot{\text{V}}}$O_2_ − 6.04 ${\dot{\text{N}}}$)^26^. The R-R intervals of the electrocardiogram were continuously monitored using a telemetric heart rate monitor (LX-3230, Fukuda Denshi Co., Ltd., Tokyo, Japan). The experiment was preceded by an adaptation night in the metabolic chamber, during which sensors of heart rate monitor (and electrodes of a polysomnographic recording system if sleep recording was scheduled in the experiment) were attached to the subjects. Prescribed diet was designed to achieve individual energy balance over the 24 h of indirect calorimetry, comprising 15% protein, 25% fat and 60% carbohydrate, expressed as percentage of total energy intake.

#### Polysomnography

Sleep was recorded polysomnographically using a PSG-1100 system (Nihon Kohden). The records were scored every 30 s to stage wakefulness (W), stage 1, stage 2, slow wave sleep (SWS), and rapid eye movement (REM) sleep according to standard criteria^8,27^.

#### Thermometry

Core body temperatures were continuously recorded using an ingestible core body temperature sensor (CorTemp, HQ Inc, Palmetto, FL, USA), 23 × 10.25 mm, weighing 2.75 g. The signal from the sensor passed through the body to the recorder, which was worn by the subjects around the stomach area. The sensor was accurate to ± 0.01 °C and was calibrated with hot water before each use^22,23^.

#### Continuous glucose monitoring

Glucose levels were continuously measured with a glucose monitor (iPro2, Medtronic MiniMed, Northridge, CA, USA). The sensor was inserted under the abdominal skin and measured interstitial glucose every 5 min. The readings were converted to blood glucose level by calibration against finger-stick blood glucose measurements 4 times a day^21^. The mean absolute relative difference value of this device was 11%^28^.

### Statistical analysis

Data are shown as mean ± standard error (SE) for groups. Differences in the physical characteristics of the subjects, energy metabolism parameters, and sleep architecture between the 2 groups were analyzed by Student’s *t* test. To compare time course of energy metabolism, blood glucose, core body temperature, heart rate and its variabilities, hourly average in each individual was calculated, analyzed using a linear mixed-models analysis of variance (ANOVA) with repeated measures and Bonferroni’s correction for multiple comparisons at various time-points between the 2 groups. To eliminate the effect of difference in body size of men and women, repeated-measure analysis of covariance (ANCOVA) was conducted to compare energy expenditure and substrate oxidation with FFM as a covariate. Correlations were assessed using the Pearson correlation coefficient. Statistical analyses were performed using SPSS statistics software (Version 26.0; IBM Corporation) for Macintosh.

## Results

### Time course of the RQ, energy metabolism, core body temperature, blood glucose and heart rate variabilities

**Figure 1:**
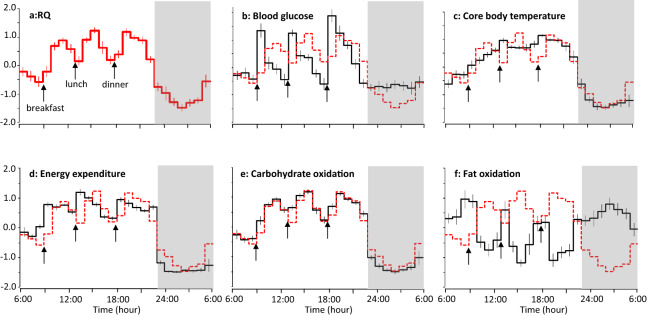
Standardized time course of energy metabolism, body temperature and blood glucose. Hourly average of RQ (**a**), blood glucose (**b**), core body temperature (**c**), energy expenditure (**d**), carbohydrate oxidation (**e**) and fat oxidation (**f**) of each subject were standardized, and mean ± SE of 11 men are shown. For comparison, time course of RQ was shown as red dotted line in panels (**b**–**f**). Data were derived from a sedentary control trial of a previous experiment focused on the effect of exercise on peripheral clock gene expression (24.5 ± 2.8 years; BMI 22.2 ± 1.9 kg/m^2^; body fat 16.1 ± 3.8%)^22^. Prescribed diet was provided as breakfast (9:00), lunch (13:00), and dinner (18:00). Subjects slept for 7 h from 23:00 to 6:00 (grey bars).

Eleven men stayed in a metabolic chamber for 24 h as a sedentary control of the experiment focused on the effect of exercise on clock gene expression^22^, in which energy metabolism, blood glucose, core body temperature, heart rate and its variability were simultaneously measured. Prescribed diet was provided as breakfast (9:00), lunch (13:00), and dinner (18:00), and energy balance over the 24 h of indirect calorimetry was + 27 ± 34 kcal/24 h. RQ and non-protein RQ increased in response to each meal, reached its nadir, and then re-ascended after midnight (Fig. 1a, Appendix Fig. 1a). To compare time course of RQ with that of core body temperature, blood glucose and energy metabolism, standardized values are shown in Fig. 1. Core body temperature, energy expenditure and carbohydrate oxidation also decreased after bedtime, followed by re-ascend after midnight, with the RQ increase being particularly salient. Fat oxidation decreased in response to each meal, increased after sleep onset but it was followed by a decrease before awakening. Time course of RQ was highly correlated to that of energy expenditure (r = 0.887, P < 0.001), carbohydrate oxidation (r = 0.976, P < 0.001), fat oxidation (r = − 0.841, P < 0.001) and core body temperature (r = 0.913, P < 0.001). Correlation of RQ with blood glucose was moderate (r = 0.573, P < 0.01).

Heart rate and sympathetic nervous system activities estimated from power ratio of low frequency to high frequency component of heart rate variability (LF/HF) were lower during sleep, but its time course during sleep was not parallel to that of RQ (Appendix Fig. 2). Parasympathetic nervous system activities estimated from high frequency component of heart rate variability (HF) increased during sleep. RQ was highly correlated to heart rate (r = 0.850, P < 0.001) and moderately correlated to HF (r = − 0.648, P < 0.001), but correlation to LF/HF was not statistically significant (P = 0.063).

### Time course of energy metabolism in metabolically flexible and inflexible subjects

**Figure 2:**
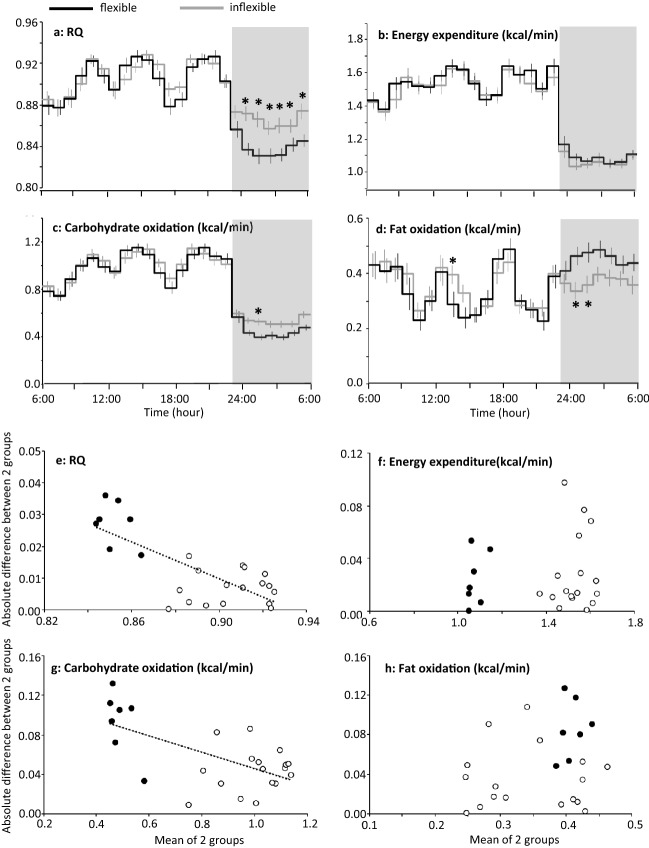
24 h profile of energy metabolism in metabolically flexible and inflexible subjects. (**a**–**d**) Forty-one young men were grouped as metabolically flexible (n = 20) or inflexible (n = 21) according to the magnitude of range of RQ over the 24 h^19–22^. Subjects took breakfast (7:00 or 9:00), lunch (12:00, 12:30 or 13:00), and dinner (18:00), and slept for 7 h (23:00–06:00, grey bars). Mean ± SE of RQ, energy expenditure, carbohydrate oxidation and fat oxidation were shown for metabolically flexible (black lines) and inflexible (grey lines) group. A linear mixed-models ANOVA showed a significant effect of time (P < 0.01) and a group × time interaction (P < 0.01) but main effect of group was not significant for RQ (P = 0.145), carbohydrate oxidation (P = 0.420) and fat oxidation (P = 0.731). For energy expenditure, effect of time was significant (P < 0.01) but main effect of group (P = 0.726) and group × time interaction (P = 0.170) was not significant. *Represents significant difference between the 2 subgroups by post hoc pair-wise comparisons (P < 0.05). (**e**–**h**) Absolute differences between metabolically flexible and inflexible subgroup were plotted against mean of the two subgroups for RQ, energy expenditure, carbohydrate oxidation and fat oxidation. Values during sleep were shown as filled symbols (filled black circles). Significant negative correlation between absolute difference and mean of the two subgroups was observed in RQ and carbohydrate oxidation (P < 0.001).

RQ increased in response to meal in the daytime and decreased during the night, which defines range of RQ over the 24 h, i.e. metabolic flexibility. In a dataset of 24 h indirect calorimetry^19–22^, 41 young men (age 21–33 years) without obesity (BMI < 30 kg/m^2^) were divided into 2 subgroups based on the magnitude of the range of RQ over the 24 h in each individual; equal or more than 0.13 in metabolically flexible (n = 20, 0.142 ± 0.002), and less than 0.13 in metabolically inflexible subgroup (n = 21, 0.102 ± 0.005). Energy balance over the 24 h of indirect calorimetry was + 44 ± 32 and + 47 ± 26 kcal/24 for flexible and inflexible group, respectively (P = 0.934). Age of the metabolically flexible and inflexible subgroups was 24.2 ± 0.6 and 24.7 ± 0.6 years, respectively (P = 0.56). Body mass index (BMI; 22.6 ± 0.4 vs 21.7 ± 0.5 kg/m^2^, P = 0.196) and body fat (16.2 ± 0.8 vs 14.8 ± 0.9%, P = 0.210) were similar between the 2 subgroups. The average RQ over the 24-h was not significantly different between the 2 subgroups (0.886 ± 0.004 vs 0.895 ± 0.004, respectively, P = 0.145). Similarly, average non-protein RQ over the 24-h was not significantly different between the 2 subgroups (0.899 ± 0.005 vs 0.909 ± 0.005, P = 0.171). Although the response to meals was similar between the 2 subgroups, RQ and non-protein RQ during sleep were lower in the subjects with metabolic flexibility than in subjects with metabolic inflexibility (Fig. 2a, Appendix Fig. 1b). Accumulated energy expenditure over the 24-h was 2046 ± 44 and 1987 ± 63 kcal/24 h for metabolically flexible and inflexible subgroup, respectively (P = 0.458), and its time course was similar between the 2 subgroups (Fig. 2b). Accordingly, metabolically flexible subgroup oxidized more fat and less carbohydrate during the night (Fig. 2c,d). There were no significant differences in time course of heart rate and its variabilities (LF/HF and HF) between the 2 subgroups (Appendix Fig. 3).

Visual inspection of the time course of RQ suggested that the difference in RQ between the 2 subgroups became clearer when RQ of both subgroups was lower; in the late afternoon and during sleep. Absolute difference of RQ between the 2 subgroups was negatively correlated with mean values of the 2 subgroups, and similar negative correlation was also observed in carbohydrate oxidation (Fig. 2e,g). On the other hand, in energy expenditure and fat oxidation, difference between the 2 subgroups was not correlated with mean of the 2 subgroups (Fig. 2f,h). Collectively, differences in energy metabolism between metabolically flexible and inflexible subgroups emerged as difference in fuel selection at midnight.

### Time course of energy metabolism in two age groups of 10 years apart

In addition to 41 men without obesity assessed for metabolically flexible and inflexible subgroups in a previous section^19–22^, another 12 male subjects aged 37.1 ± 4.3 years (range 20–56) without obesity^9^ were incorporated for statistical analysis. Total of 53 men were divided based on their age; under 25 years old (n = 27, age 22.5 ± 0.2 years, body weight 67.2 ± 1.7 kg, BMI 22.2 ± 0.4 kg/m^2^, body fat % 16.2 ± 0.8) and 25 years or more (n = 26, 32.5 ± 2.0 years, 65.0 ± 1.9 kg, 21.9 ± 0.3 kg/m^2^, 16.2 ± 0.7%). Body weight (P = 0.382), BMI (P = 0.581) and body fat (P = 0.949) were not significantly different between the two age groups. Energy balance over the 24 h of indirect calorimetry was + 40 ± 26 and + 57 ± 21 kcal/24 h for younger and older group, respectively (P = 0.620). The average RQ over the entire calorimetry was not significantly different between the 2 age groups (0.886 ± 0.003 vs 0.898 ± 0.006 for younger and older group, respectively; P = 0.078). Similarly, average non-protein RQ over the entire calorimetry was not significantly different between the 2 age groups (0.901 ± 0.004 vs 0.912 ± 0.0.007, for younger and older group, respectively, P = 0.176).

**Figure 3:**
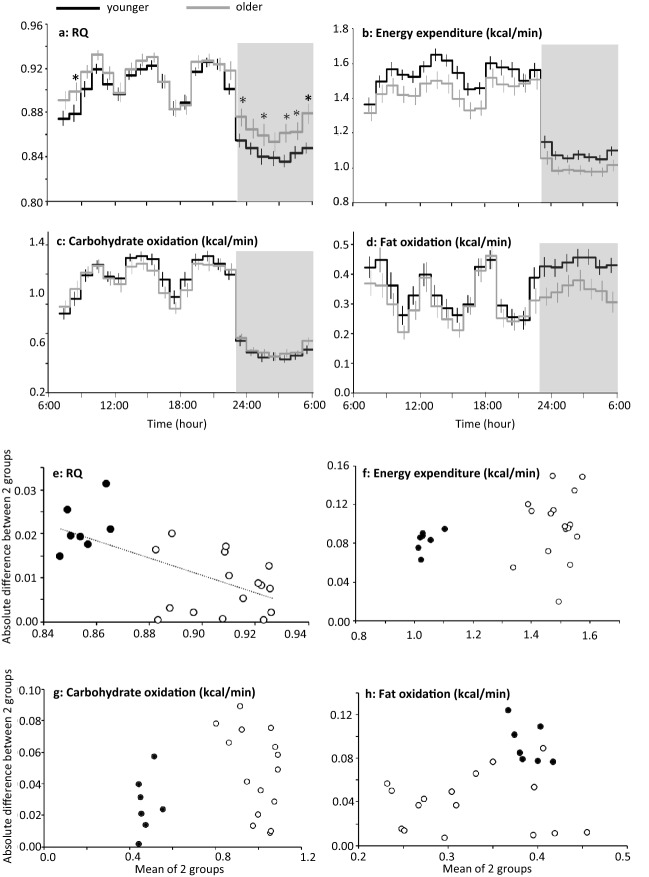
Time course of energy metabolism in two age groups of 10 years apart. (**a**–**d**) Fifty-three men were grouped as younger (n = 27, under 25 years of age) or older (n = 26, 25 years or more)^9,19–22^. Subjects took breakfast (7:00, 8:00 or 9:00), lunch (12:00, 12:30 or 13:00), and dinner (18:00), and slept for 7 or 8 h from 23:00 (grey bars). Because of unequal duration of sleeping period, the 8th hour of sleep in one experiment was not included for statistical analysis^9^. Mean ± SE of RQ, energy expenditure, carbohydrate oxidation and fat oxidation for 23 h common to all data sets were shown for younger (black lines) and older (grey lines) group. A linear mixed-models ANOVA showed a significant effect of time (P < 0.01) and a group × time interaction (P = 0.032), but main effect of group was not significant (P = 0.078) for RQ. Similarly for carbohydrate oxidation, significant effect of time (P < 0.01) and a group × time interaction (P = 0.043) were found, but main effect of group was not significant (P = 0.792). For energy expenditure and fat oxidation, main effect of time was significant (P < 0.01), but main effect of group (P = 0.066 for energy expenditure and P = 0.129 for fat oxidation) and group × time interaction was not significant (P = 0.376 and P = 0.290). *Represents significant difference between the 2 subgroups by post hoc pair-wise comparisons (P < 0.05). Significant difference in RQ in the morning requires cautious interpretation, since % of subjects who took breakfast before 9:00 was not matched; 52% in younger and 69% in older group, respectively. (**e**–**h**) Absolute difference between two age groups were plotted against mean of the two subgroups for RQ, energy expenditure, carbohydrate oxidation and fat oxidation. Values during sleep were shown as filled symbols (filled blue circles). Significant negative correlation between absolute difference and mean of the two subgroups was observed in RQ (P < 0.001).

Compared to younger group, RQ and non-protein RQ were higher in older group during sleep (Fig. 3a, Appendix Fig. 1c). Among all of 53 subjects, nadir of RQ was positively correlated to age (r = 0.331, P = 0.0156), but not significantly related to body weight (P = 0.272), BMI (P = 0.709) or body fat % (P = 0.769). Average energy expenditure over the 23 h in older group (1.30 ± 0.04 kcal/min) was slightly lower than that of younger group (1.40 ± 0.03 kcal/min) although the difference was not statistically significant (P = 0.066) (Fig. 3b). There was no significant difference in substrate oxidation (Fig. 3c,d). Significant negative correlation between absolute difference and mean of the two subgroups was observed in RQ (P < 0.001), but not in energy expenditure and substrate oxidation (Fig. 3e–h). Heart rate and autonomic nervous system activity were not statistically different between the two age groups (Appendix Fig. 4).

### Twenty-four-hour time course of energy expenditure and RQ in young men and women

RQ and core body temperature follow U-shaped time course during sleep. Since a sex difference exists in the time course of body temperature during sleep^11–13^, we compared time course of sleeping energy metabolism between men and women. Men (age 23.9 ± 1.4 years; BMI 22.1 ± 1.6 kg/m^2^) and women (age 23.9 ± 3.8 years; BMI 22.3 ± 1.8 kg/m^2^) stayed in the metabolic chamber following an identical experimental protocol as the sedentary control of the experiment focused on the effect of exercise on 24 h fat oxidation^18,19^. Prescribed diet was provided as breakfast (8:00), lunch (12:00), and dinner (18:00). The contributions of breakfast, lunch, and dinner to the total 24-h energy intake were 33%, 33%, and 34%, respectively. Energy balance was + 127 ± 28 and + 122 ± 34 kcal/24 h for men and women, respectively (P = 0.894). Subjects slept for 7 h from 23:00 to 6:00. The women participated in the study during the follicular phase.

**Figure 4:**
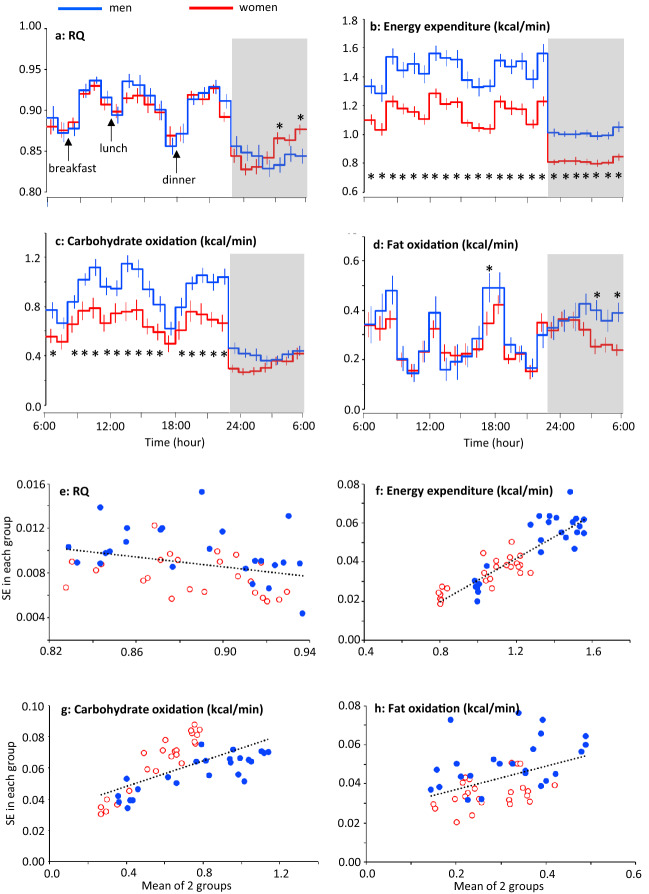
24-h energy metabolism in men and women. (**a**–**d**) Mean ± SE of RQ (**a**), energy expenditure (**b**), carbohydrate oxidation (**c**) and fat oxidation (**d**) in 10 men (blue lines)^19^ and 9 women (red lines)^18^ were calculated from a sedentary trial in previous experiments focused on the effect of exercise on 24-h fat oxidation. Prescribed diet was provided as breakfast (8:00), lunch (12:00), and dinner (18:00). Subjects slept for 7 h from 23:00 to 6:00 (grey bars). A linear mixed-models ANOVA showed a significant effect of time (P < 0.01), and a group × time interaction (P < 0.01) for RQ, energy expenditure, carbohydrate oxidation and fat oxidation. Main effect of group was significant for energy expenditure and carbohydrate oxidation (P < 0.01), but that for RQ (P = 0.924) and fat oxidation (P = 0.461) was not significant. *Represents significant difference between the 2 subgroups by post hoc pair-wise comparisons (P < 0.05). (**e**–**h**) Relation between the mean and SE of RQ (**e**), energy expenditure (**f**), carbohydrate oxidation (**g**) and fat oxidation (**h**) of men (blue filled circles) and women (red open circles). Negative correlation in RQ and positive correlation in energy expenditure, carbohydrate oxidation and fat oxidation were observed between the mean and SE.

The average RQ over the 24 h was similar between men (0.887 ± 0.009) and women (0.886 ± 0.006, P = 0.924), and average non-protein RQ over the 24 h was also not significantly different between men (0.904 ± 0.009) and women (0.901 ± 0.008, P = 0.831). In both men and women, RQ and non-protein RQ decreased during sleep, and began to increase prior to awakening. The increase in the RQ during sleep seemed to be earlier in women than in men, and RQ at 5th and 7th hour of sleep was significantly higher in women than that in men (Fig. 4a, Appendix Fig. 1d). Energy expenditure, and substrate oxidation were higher in men than women reflecting the difference in their body size (67.2 ± 2.8 vs 57.8 ± 1.6 kg) (Fig. 4b–d). However, when adjusted with fat free mass, there was no significant main effect of sex on energy expenditure (P = 0.873), carbohydrate oxidation (P = 0.808) and fat oxidation (P = 0.831). There were no significant differences in heart rate and its variability between men and women (Appendix Fig. 5).

We noticed that individual variation, reflected as SE in each group, changed throughout the day. Standard error of RQ became larger when mean value is low; after overnight fasting before breakfast, in the late afternoon and during sleep. Multiple regression analysis with sex and mean values as independent variables revealed that the SEs of RQ were negatively correlated with means (P = 0.014), i.e. individual variation became clearer when RQ is low (Fig. 4e). This observation is in contrast with positive correlations between mean and SE in energy expenditure and substrate oxidation (P < 0.01) (Fig. 4f–h).

### Nadir of the RQ and energy expenditure during sleep in men and women

The U-shaped time course during the night were observed in RQ, and the increase in the RQ during sleep seemed to be earlier in women than in men. The time between bedtime and the nadir of the RQ (5-min period of the lowest value) was compared between men and women by applying statistical rigor to our database of sleeping energy metabolism. Data for 79 men and 36 women were collected from our previous studies with 24 h indirect calorimetry^18–22^ and indirect calorimetry over an entire sleeping period^8,23–25^. Pooled data for the women included indirect calorimetry during the follicular (n = 20) and luteal (n = 10) phases, and cases without a record of the subjects’ menstrual cycle (n = 6). The nadir of RQ was observed significantly earlier in women than in men, while the nadir of energy expenditure was observed significantly later in women than in men (Table 2). Time course of core body temperature was available for 30 men and 18 women^21–24^, and nadir was observed significantly earlier in women than in men. Time between bedtime and nadir of RQ was correlated to that of core body temperature (r = 0.368, P < 0.01), but not to that of energy expenditure (P = 0.192).

**Table 2:** Nadir of body temperature, RQ and energy expenditure during sleep in men and women.

	Men	Women	P-value
RQ^a^	3.36 ± 0.19	1.95 ± 0.30	0.0001
Energy expenditure^a^	3.72 ± 0.22	4.80 ± 0.33	0.0076
Core body temperature^b^	3.48 ± 0.33	1.98 ± 0.49	0.0115

Time of the nadir (5-min period of the lowest value) was shown as time after bedtime in hours for RQ, energy expenditure and body temperature. Bedtime of each experiment was 23:00^18–22^ or at subjects’ habitual bedtime (23:00–24:30)^8,23–25^. In studies with an 8-h sleep opportunity, data of the first 7 h was included for statistical analysis^8,23–25^. The nadir of the RQ (P = 0.608), energy expenditure (P = 0.858) and body temperature (P = 0.310) during sleep in women was not related to their menstrual cycle.

^a^Mean age of the men (n = 79, 24.5 ± 0.5 years) and women (n = 36, 23.4 ± 0.3 years) were comparable (P = 0.169)^8,18–25^.

^b^Mean age of the men (n = 30, 23.8 ± 0.3 years) and women (n = 18, 23.7 ± 0.5 years) were comparable (P = 0.750)^21–24^.

### Sleep architecture and the RQ

**Figure 5:**
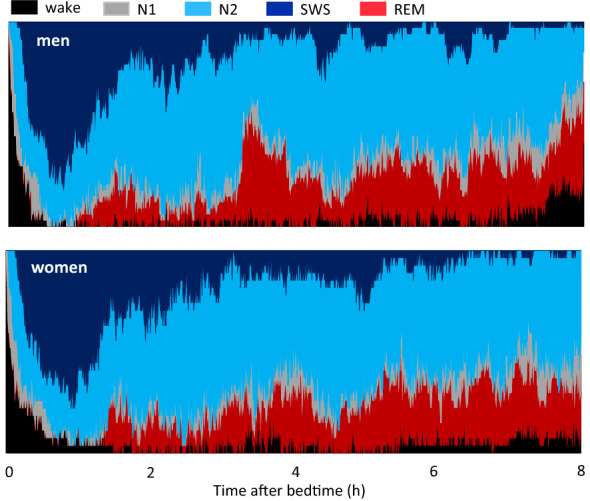
Cumulative display of sleep architecture during simultaneous assessment of energy metabolism. The percentage of subjects in each sleep stage is shown for men (upper panel) and women (lower panel). The total number of subjects was 38 men and 27 women (11 in follicular phase, 11 in luteal phase, and 5 cases without record). Subjects slept for 8 h in a metabolic chamber for indirect calorimetry^8,23–25^. In this dataset, the nadir of the RQ was 3.00 ± 0.26 and 2.04 ± 0.39 h after bedtime for the men and women, respectively (P < 0.0368).

Differences in RQ between metabolically flexible and inflexible subgroups emerged at midnight, and there was a sex difference in time course of RQ during the night. These observations led us to relate metabolic data with sleep architecture. Sleep architecture, evaluated as total time and latencies, was similar between men (n = 34) and women (n = 27: 11 follicular, 11 luteal, and 5 cases without record) (Table 3)^8,23–25^. Sleep followed characteristic cyclic changes in the sleep stage, termed the sleep cycle; after the first sleep cycle, SWS gradually decreased and was replaced by REM sleep in men and women (Fig. 5). Despite the similar sleep architecture between men and women in this dataset, nadir of RQ after bedtime was observed significantly earlier in women (2.04 ± 0.39 h) than in men (3.00 ± 0.26 h; P = 0.0368).

**Table 3:** Comparison of sleep architecture between men and women.

	Men	Women	P-value
Age	23.3 ± 0.4	23.6 ± 0.5	0.615
Total bedtime, min	480 ± 0	480 ± 0	
Total sleep time, min	452 ± 3	447 ± 5	0.371
Wakefulness, min	26 ± 3	26 ± 5	0.934
Sleep latency, min	8 ± 1	9 ± 2	0.743
Sleep efficiency, %	94 ± 1	93 ± 1	0.455
Stage 1, min	45 ± 3	42 ± 5	0.601
Stage 2, min	240 ± 6	236 ± 8	0.635
SWS, min	83 ± 6	88 ± 8	0.609
REM sleep, min	84 ± 3	81 ± 4	0.507
REM sleep latency, min	116 ± 8	112 ± 7	0.702

Values are means ± SE for men (n = 34) and women (n = 27)^8,23–25^.

## Discussion

### Limitations of the present study

We retrospectively analyzed the data of previous studies. Temperature and relative humidity in the metabolic chamber, type of gas analyzer and algorithm to calculate ${\dot{\text{V}}}$O_2_ and ${\dot{\text{V}}}$CO_2_ were same. In all studies, subjects took dinner 5 h before bedtime, but details of experimental protocol were not identical: differences in time of breakfast (7:00 or 9:00) and lunch (12:00, 12:30 or 13:00) and time in bed (7 h or 8 h). Polysomnographic recording of sleep, thermometry and continuous glucose monitoring were not performed in every study. A key aspect of the present study is that it is based on indirect calorimetry in adults without obesity. Obviously, additional data of subjects with obesity or diabetes helps to understand pathogenesis of metabolic inflexibility, insulin resistance and diabetes.

### Interpretation of the time course of the RQ during sleep

Fuel selection in the body is estimated from the RQ, i.e., the ratio of CO_2_ production to O_2_ consumption. RQ is a dimensionless quantity, allowing for comparison between subjects of different body sizes such as men and women. A higher RQ implies carbohydrate oxidation whereas a lower RQ reflects fat oxidation. To focus on the selection between carbohydrate and fat as substrate for oxidation, non-protein RQ is preferred rather than RQ. However, there is one caveat that time resolution of urinary nitrogen excretion, with which non-protein RQ was calculated, is low. In the present study, ${\dot{\text{V}}}$CO_2_ and ${\dot{\text{V}}}$O_2_ were calculated every 5 min, but urinary nitrogen excretion was assumed to be constant during the indirect calorimetry. Time course of non-protein RQ (Appendix Fig. 1) confirmed the main finding of the present study: U-shaped time course during sleep (Fig. 1), difference between metabolically flexible and inflexible subjects (Fig. 2), effect of age (Fig. 3) and sex (Fig. 4).

On the whole, RQ during sleep is lower than that when awake, and this is underscored by reduced levels of glucose and insulin during sleep^29^. A closer look at the time course of the RQ during sleep, however, revealed that RQ begins to increase prior to awakening. During prolonged fasting, gluconeogenesis from alanine (RQ = 0.13) and ketogenesis (RQ = 0.57) may play a role to support energy metabolism, but the RQ of these metabolic pathways is low^26^. Protein oxidation is estimated from the urinary excretion of nitrogen, and it is possible that protein catabolism and oxidation increase as fasting continues. The RQ of protein oxidation, however, is ~ 0.81^26^. The increase in RQ above this level, therefore, cannot be attributed to increased protein oxidation. Collectively, the time course of the RQ during sleep suggests that oxidized substrates shift from fat to carbohydrate before awakening in a sex-specific manner, despite prolonged fasting.

### Sleep and RQ

A circadian component of energy metabolism was demonstrated by a forced desynchrony protocol, in which subjects experienced a 3-week 28-h rest-activity schedule^14^. Repeated measures of resting energy expenditure after each “overnight” fast allowed for reconstruction of the endogenous circadian component uncoupled from sleep–wake and activity-related effects. The nadir of RQ was in the late biologic evening (21:00), earlier than we observed during 24 h indirect calorimetry. The amplitude of the circadian component of the RQ assessed by cosinor regression was 0.012, smaller than the diurnal change in the RQ in the present study: ~ 0.05. More importantly, the identity of the mechanisms underlying circadian changes in the RQ is not known.

Along with cyclic transitions of sleep stages, the dominant sleep stage gradually shifts from SWS to stage 2 and REM sleep over the entire sleeping period, and there are short periods judged as wake based on electroencephalography, i.e. wake after sleep onset (Fig. 5). In our previous study, the time course of energy metabolism during sleep was decomposed into the effect of sleep stages and the effect independent of sleep stages, i.e., the effect of time after sleep onset^8^. Reflecting various physiologic changes during REM sleep and wake after sleep onset, energy expenditure during these sleep stages was higher than that during SWS. However, the differences in the RQ among sleep stages were not significant^8^. The findings from simultaneous assessment of sleep and energy metabolism in the present study were as follows. First, an increase in the RQ was observed during the night while confirming normal sleep patterns through electroencephalogram recordings. This excluded possible experimental artifacts, such as difficulties in maintaining sleep in the experimental setting, affecting sleeping energy metabolism. Second, sex differences in the sleep architecture are reported in some studies^15,16^, although another study found no differences in the sleep architecture between sexes^30^. In the present study, sleep architecture was similar between men and women, whereas the time of the nadir of the RQ during sleep was significantly earlier in women than in men. Therefore, the sex-specific time difference in the increase in the RQ before awakening is not likely related to the differences in sleep architecture between men and women.

### Mechanism(s) underlying the regulation of the RQ during sleep in a sex-specific manner

Energy homeostasis depends on the substrate supply and demand^7^, the response to which is manifested as metabolic flexibility. A number of studies measured circulating levels of metabolites during sleep. Despite prolonged fasting, blood glucose levels remain stable during sleep^29^. On the other hand, free fatty acid (FFA) levels peak in the middle of the sleep period and decline again toward the beginning of the next wake period^29^. This transient increase in circulating FFA may underlie the increase in its oxidation in the middle of the night, i.e., the U-shaped time course of the RQ during sleep. Sex differences in the time course of circulating FFA have not been evaluated. Among lipolytic and antilipolytic hormones, the levels of epinephrine, norepinephrine, and insulin remain low during sleep^29,31^. Although growth hormone levels are upregulated during sleep, endogenous growth hormone plays a very limited metabolic role to stimulate lipolysis during the daily feed/fast cycle^32^, and the amplitude and acrophase for plasma growth hormone levels do not differ between men and women^33^. The plasma adrenocorticotropic hormone (ACTH) concentration increases during the second half of sleep^34^, ruling out a possible causal link between ACTH secretion and elevated circulating FFA.

The time course of the RQ during sleep shares common features with that of core body temperature, which reaches its nadir at midnight and begins to increase prior to awakening. Time course of RQ and core body temperature was highly correlated, and time of the nadir of these two variables during sleep was significantly correlated. These observations suggest that both rhythms are under control of the same endogenous circadian pacemaker. Core body temperature is regulated by an endogenous pacemaker in the hypothalamic suprachiasmatic nuclei^35^. The suprachiasmatic nuclei also drives the circadian rhythm of melatonin and cortisol, both of which are considered reliable markers of the master clock of the body^36,37^. Interestingly, the circadian rhythm of melatonin, but not of cortisol, shows a sex difference. Melatonin secretion begins and ends earlier in women than in men^12,13^. A recent human study reported that the RQ in the evening was negatively correlated with salivary melatonin (r = − 0.76)^38^. Melatonin is not categorized as a classical lipolytic hormone. The physiologic concentration of melatonin stimulates lipolysis in porcine and bovine intramuscular adipocytes^39,40^, but inhibits lipolysis in rat inguinal adipocytes in a site-specific manner^41^. Discrepancies in the effect of melatonin on lipolysis seem to be related to differences between diurnal (porcine, bovine) and nocturnal animals (rat).

Sex steroids are obvious mechanistic candidates underlying the time course of the RQ in a sex-specific manner. Despite dynamic changes in estrogen and progesterone levels during the menstrual cycle, the time of the nadir in the RQ is comparable between the follicular and luteal phases^23^, and earlier in women than in men in the present study. An earlier timing of body temperature rhythm changes relative to the sleeping period in women compared with men was reported in young^12^ and older subjects^42^, although postmenopausal women presumably had lower circulating ovarian steroid levels. Testosterone increases during sleep^43^, but an acute effect of testosterone has little effect on either serum FFA or the RQ^44^. Therefore, ovarian and testicular steroids are unlikely to be directly involved in upregulating the RQ during sleep. The concentrations of other hormones also change at midnight; leptin peaks and cortisol begin to increase at midnight^34^. Leptin, which stimulates glucose oxidation, declines when the RQ begins to increase during the second half of the sleeping period. Cortisol increases blood glucose levels through gluconeogenesis, but its effect on glucose oxidation to increase the RQ is not established. Of note, there is no sex difference in the phase angle of the circadian rhythm of leptin^45^ or in the cortisol concentration^37^. Involvement of autonomic nervous system for regulation of RQ during sleep is unlikely, because there was no significant difference in time course of heart rate variabilities (LF/HF and HF) between metabolically flexible and inflexible subgroups, different age groups, and men and women (Appendix Figs. 3–5). To our knowledge, melatonin is the only hormone that meets the requirement to explain time course of RQ during sleep; association with a decreased RQ during sleep, and a sex difference in its diurnal rhythm.

According to the above discussion, the diurnal rhythm of the RQ comprises several components. First, changes in the nutritional state, from fed to fasted, set the tone for a gradual decrease in the RQ during sleep. Second, the circadian component of energy metabolism and/or nocturnal melatonin secretion transiently decrease RQ during sleep in a sex-specific manner. As the third unidentified factor, homeostatic mechanisms regulating sleep may underlie the U-shaped time course of the RQ during sleep. A large number of substances tested for their effects on wakefulness and sleep have effects on hunger, satiety, and energy metabolism^46^. Neurosubstances, including orexin, serotonergic substances, insulin, leptin, neuropeptide Y, interleukin-6, and bombesin, have multiple roles in sleep and energy metabolism. It is plausible that actions of these neurosubstances change as sleep drive decreases after sleep onset, and that energy metabolism is affected in turn. The relation between the actions of these neurosubstances and sleeping energy metabolism remains to be addressed in human studies. Of note, we observed that an orexin receptor antagonist induces sleep, modifies sleep architecture, and suppresses energy expenditure^27^.

### Inter-individual variations in the RQ during sleep and its physiologic relevance

As a working hypothesis, it has been proposed that certain characteristics of energy metabolism, which include the RQ, may precede the obese state and contribute to its development^47^. Subjects who are in energy balance are also in substrate balance; the RQ measured over the 24 h period is equal to the food quotient, the theoretical RQ produced by the diet^48^. As a consequence, 24-h RQ assessed under a weight-stable and diet-controlled condition is not a predictor of future weight gain^49,50^. Even when the energy balance is maintained over the 24 h, the nutritional state alternates between postprandial and postabsorptive. Inflexibility in adjusting the RQ to transient changes in the nutritional state within a day may be a metabolic characteristic that precedes an obese state. Considering the findings of the present study in young subjects without obesity in the context of the pathogenesis of metabolic syndrome, it is noteworthy that difference in RQ between metabolically flexible and inflexible subgroup became significant only during sleep without noticeable differences in RQ after overnight fasting or the response of the RQ to meal consumption (Fig. 2a). Individual difference in RQ became prominent when the RQ was lower during sleep (Fig. 2e). The strong effects of meal consumption on blood glucose and subsequent insulin secretion increase the RQ and mask individual differences during the daytime. Similarly, the effect of 10 years difference in age and sex difference in time course of RQ became significant during sleep (Figs. 3a, 4a). Thus, inter-individual variations of RQ expands at midnight, and sleeping RQ might serve as a window to gain insight into the early-stage pathogenesis of metabolic inflexibility. It is of note that average energy expenditure over the 23 h in older group was slightly lower than that of younger group although the difference was not statistically significant (Fig. 3b). This 7% decrease in energy expenditure over a decay coincides with sum of 3.3% difference in body weight in the present data set (67.2 ± 1.7 kg for younger and 65.0 ± 1.9 kg for older group) and presumed 3.5% decrease in basal metabolic rate per decade^51^. Decrease in energy expenditure over a decay was not detected with statistical significance, while blunted decrease of RQ during sleep was detected in the present study.

According to the difference between 24-h and sleep RQ, Mynatt et al. classified subjects as metabolically flexible (8 men and 8 women) and metabolically inflexible (7 men and 8 women)^6^. BMI, homeostatic model assessment of insulin resistance, and mean RQ over the 24 h were similar between the 2 groups, but subjects with metabolic inflexibility had a higher sleep RQ (0.90 ± 0.03) relative to subjects with metabolic flexibility (0.84 ± 0.08). Analysis of global skeletal muscle gene expression revealed that transcripts regulated by the RNA binding protein HuR were enriched in metabolically flexible subjects. Silencing HuR in human myotubes induced a metabolically inflexible phenotype, suggesting a role for HuR as a regulator of metabolic flexibility in skeletal muscle metabolism. Thus, the lower amplitude of the 24-h RQ rhythm due to elevated nocturnal values is an early-stage phenotype of metabolically inflexible individuals.

### Future directions

Based on the data collected using a whole-room indirect calorimetry, the present study showed that sleeping energy metabolism is not simply the result of prolonged fasting. The observed sex difference in the time course of RQ during sleep narrowed down the possible mechanisms underlying the upregulation of glucose oxidation during the latter part of the sleeping period, and suggests a possible role of melatonin. Exogenous melatonin lowers the body temperature and promotes sleep in humans^52,53^, but elevates body temperature and increases the activity level and waking in nocturnal mammals^54,55^. Together, these lines of thought open avenues for further investigations of sleeping energy metabolism in humans by monitoring melatonin levels or intervening with melatonin secretion.

Inter-individual variability in the time course of the sleeping RQ may be an upstream event of the cascade that leads to obesity, diabetes, and metabolic diseases. To examine the components of the metabolic flexibility, Galgani et al. assessed the ability to adapt fuel oxidation to fuel availability; ∆RQ adjusted for glucose infusion rate during a hyperinsulinemic euglycemic clamp, and ∆RQ adjusted for changes in β-hydroxybutyrate during prolonged fast^56,57^. Time course of plasma β-hydroxybutyrate and FFA during sleep was not available in the present study, and remained to be evaluated. One of the promising future directions is combining indirect calorimetry with omics studies to reveal the physiologic mechanism underlying individual differences in metabolic flexibility. Tissue samples provide valuable information for clarifying the mechanism underlying individual variations in the RQ. Global skeletal muscle gene expression profiles by Mynatt et al. suggest a role of the RNA binding protein HuR underlying individual differences in the sleeping RQ^6^. Interestingly, in male mice, but not female mice, with skeletal muscle-specific knockout of the HuR-encoding gene exhibit metabolic inflexibility, with mild obesity, impaired glucose tolerance, and impaired fat oxidation, compared with control littermates. Sexual dimorphism in the role of HuR remains to be studied. Metabolome analysis of urine and blood samples should be seriously considered. Particularly, urine samples are routinely collected to assess urinary nitrogen excretion, and taking advantage of this information would be a practical approach.

Another potential direction for future studies is an analysis of energy metabolism in larger and more heterogeneous populations. The present study was based on 127 recordings of indirect calorimetry in adults without obesity and the majority of the subjects were in their 20 s. The primary focus of these studies was not on metabolic flexibility or the time course of the RQ during sleep, but analysis of pooled data provided insight into the pathogenesis of the early stage of metabolic inflexibility. An international effort to set up a database for energy expenditure assessed by a doubly-labeled water method, the gold standard method for measuring energy expenditure in a free-living condition, has launched^58^. Why not pool the data of the other branches of indirect calorimetry, such as whole-room indirect calorimetry? A guidance to ensure consistency and facilitate meaningful comparisons of human energy metabolism studies across publications, laboratories, and clinical sites has recently been proposed^59^.

## Supplementary Information

Supplementary Figure 1.

Supplementary Figure 2.

Supplementary Figure 3.

Supplementary Figure 4.

Supplementary Figure 5.

Supplementary Legends.
