# Supplementary material for: Metabolic flexibility during sleep
Source: Sci Rep. 2021 Sep 8;11:17849. doi: 10.1038/s41598-021-97301-8 (PMC8426397; doi:10.1038/s41598-021-97301-8)
Supplement: Supplementary file 1 — Supplementary Figure 1. [file 41598_2021_97301_MOESM1_ESM.pptx]

## Slide 1
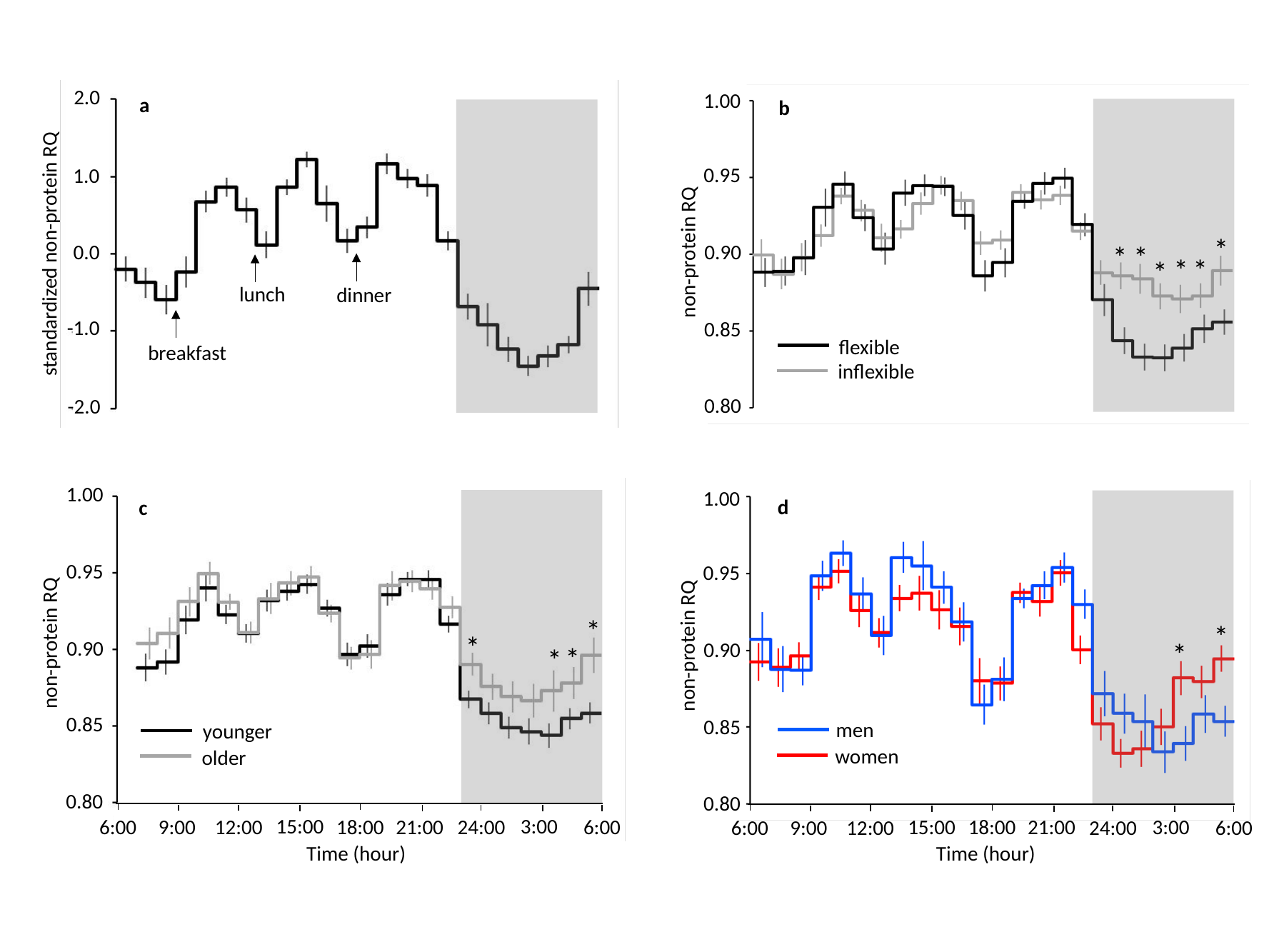

2.0
1.0
0.0
-1.0
-2.0
a
standardized non-protein RQ
lunch
dinner
breakfast
1.00
0.95
0.90
0.85
0.80
b
*
*
*
non-protein RQ
*
*
*
flexible
inflexible
1.00
0.95
0.90
0.85
0.80
c
*
*
non-protein RQ
*
*
younger
older
3:00
15:00
21:00
18:00
24:00
6:00
9:00
6:00
12:00
Time (hour)
1.00
0.95
0.90
0.85
0.80
d
*
non-protein RQ
*
men
women
3:00
15:00
21:00
18:00
24:00
6:00
9:00
6:00
12:00
Time (hour)
