# Supplementary figures and images for: Metabolic flexibility during sleep
Source: Sci Rep. 2021 Sep 8;11:17849. doi: 10.1038/s41598-021-97301-8 (PMC8426397; doi:10.1038/s41598-021-97301-8)

## Slide 1
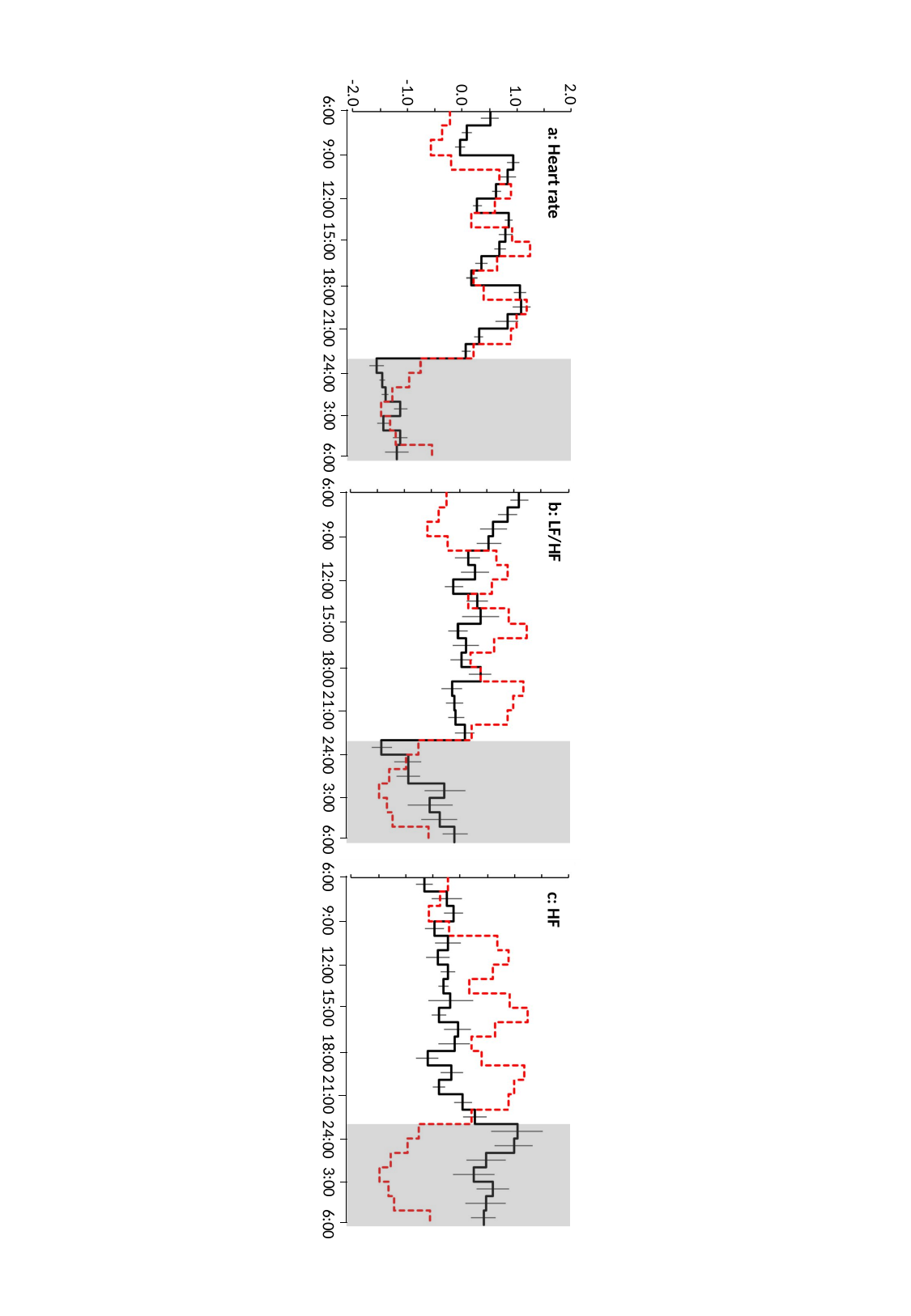

2.0
a: Heart rate
b: LF/HF
c: HF
1.0
0.0
-1.0
-2.0
3:00
15:00
21:00
18:00
24:00
6:00
9:00
6:00
12:00
3:00
15:00
21:00
18:00
24:00
6:00
9:00
6:00
12:00
3:00
15:00
21:00
18:00
24:00
6:00
9:00
6:00
12:00
b: LF/HF
a: Heart rate

Supplement: Supplementary file 2 — Supplementary Figure 2. [file 41598_2021_97301_MOESM2_ESM.pptx]

## Slide 1
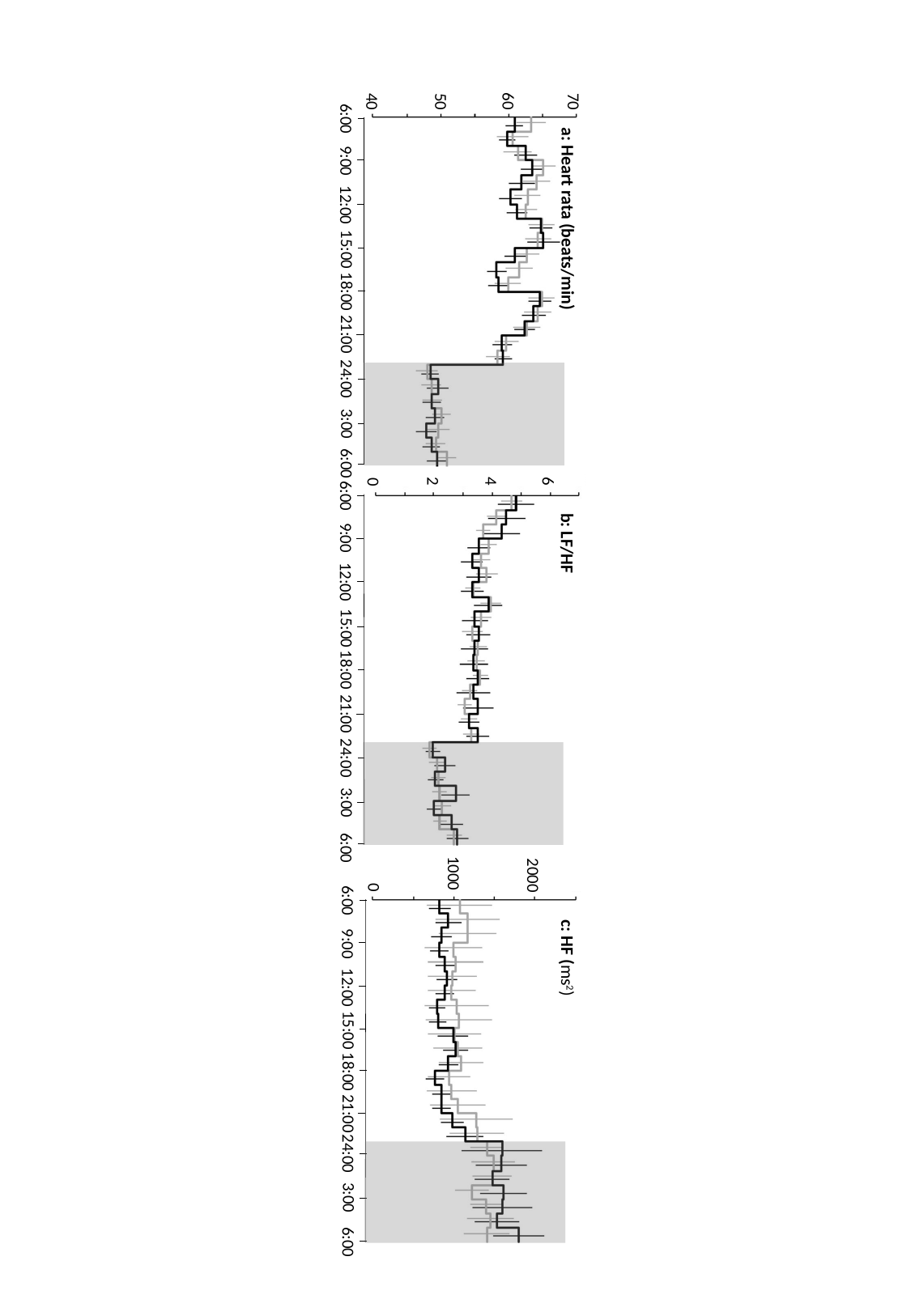

70
a: Heart rata (beats/min)
c: HF (ms2)
b: LF/HF
6
2000
60
4
1000
50
2
0
0
40
3:00
15:00
21:00
18:00
24:00
6:00
3:00
15:00
21:00
18:00
24:00
6:00
9:00
6:00
12:00
9:00
6:00
12:00
3:00
15:00
21:00
18:00
24:00
9:00
6:00
12:00
6:00

Supplement: Supplementary file 3 — Supplementary Figure 3. [file 41598_2021_97301_MOESM3_ESM.pptx]

## Slide 1
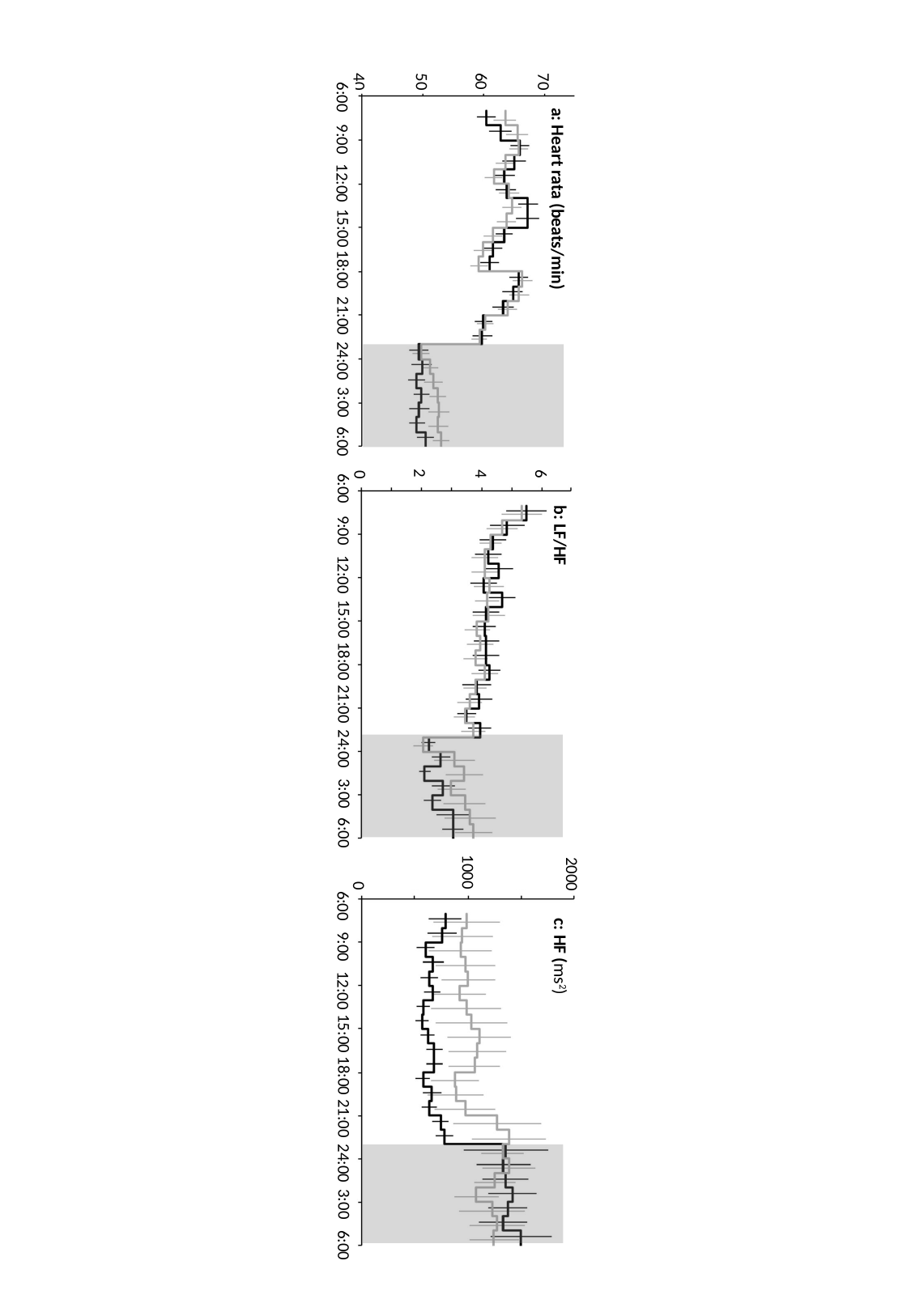

2000
b: LF/HF
c: HF (ms2)
a: Heart rata (beats/min)
70
6
60
4
1000
50
2
0
40
0
3:00
15:00
21:00
18:00
24:00
6:00
9:00
6:00
12:00
3:00
15:00
21:00
18:00
24:00
6:00
9:00
6:00
3:00
12:00
15:00
21:00
18:00
24:00
6:00
9:00
6:00
12:00

Supplement: Supplementary file 4 — Supplementary Figure 4. [file 41598_2021_97301_MOESM4_ESM.pptx]

## Slide 1
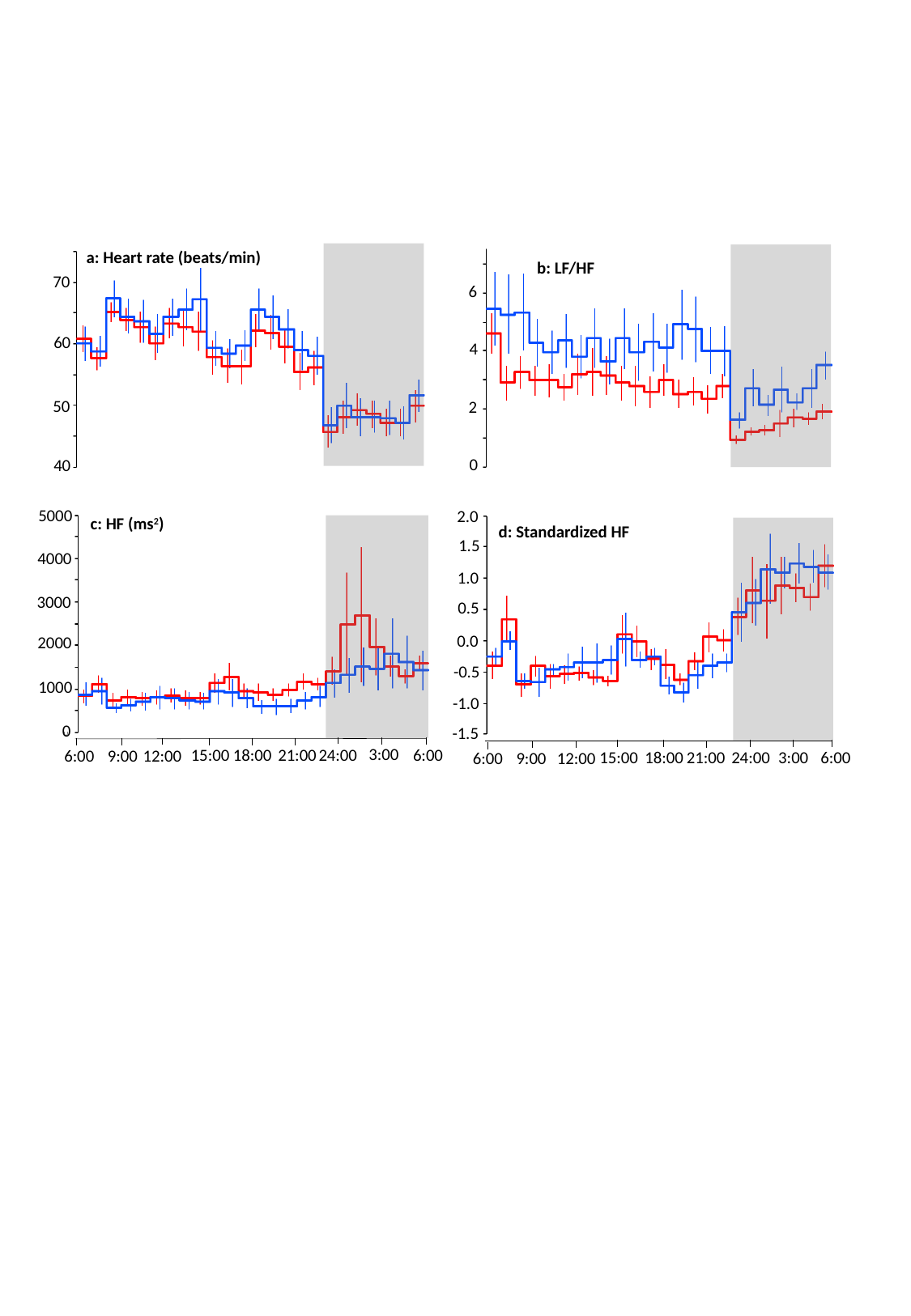

a: Heart rate (beats/min)
b: LF/HF
70
6
60
4
50
2
0
40
5000
2.0
c: HF (ms2)
d: Standardized HF
1.5
4000
1.0
3000
0.5
0.0
2000
-0.5
1000
-1.0
0
-1.5
3:00
15:00
21:00
18:00
24:00
6:00
9:00
6:00
12:00
3:00
15:00
21:00
18:00
24:00
6:00
9:00
6:00
12:00

Supplement: Supplementary file 5 — Supplementary Figure 5. [file 41598_2021_97301_MOESM5_ESM.pptx]
