# Supplementary material for: Metabolic flexibility during sleep
Source: Sci Rep. 2021 Sep 8;11:17849. doi: 10.1038/s41598-021-97301-8 (PMC8426397; doi:10.1038/s41598-021-97301-8)
Supplement: Supplementary file 6 — Supplementary Legends. [file 41598_2021_97301_MOESM6_ESM.docx]

Appendix Figure 1. 24 h profile of on-protein RQ.

**a:** Hourly average of non-protein RQ were standardized, and mean ± SE of 11 non-obese men were shown.**^22^** Subjects slept for 7 h from 23:00 to 6:00. **b:** Forty-one young men were grouped as metabolically flexible (n=20) or inflexible (n=21) according to the magnitude of range of hourly RQ over the 24 h.**^19-22^** Mean ± SE of non-protein RQ were shown for metabolically flexible (
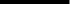
 ) and inflexible (
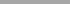
 ) group. According to a linear mixed-models ANOVA, effect of group was not statistically significant (P = 0.171), but effect of time (P<0.001) and a group x time interaction (P<0.001) were significant. *Represents significant difference between the 2 subgroups by post hoc pair-wise comparisons (P < 0.05). **c:** Fifty-three men were grouped as younger (n=27, under 25 years of age) or older (n=26, 25 years or more).**^9,19-22^** Because of unequal duration of sleeping period, the 8^th^ hour of sleep in one experiment was not included for statistical analysis.**^9^** Mean ± SE of non-protein RQ were shown for younger (
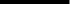
) and older (
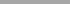
) group. According to a linear mixed-models ANOVA, effect of group was not statistically significant (P = 0.176), but effect of time (P<0.001) and a group x time interaction (P<0.01) were significant. *Represents significant difference between the 2 subgroups by post hoc pair-wise comparisons (P < 0.05). **d:** Mean ± SE of non-protein RQ in 10 men (
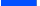
)**^19^** and 9 women (
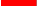
)**^18^** were calculated from a sedentary trial in previous experiments focused on the effect of exercise on 24-h fat oxidation. According to a linear mixed-models ANOVA, effect of group was not statistically significant (P = 0.831), but effect of time (P<0.001), and a group x time interaction (P<0.01) were significant. *Represents significant difference between the 2 subgroups by post hoc pair-wise comparisons (P < 0.05).

Appendix Figure 2. Standardized time course of heart rate and autonomic nervous system function.

Hourly average of heart rate (a), sympathetic nervous system activity, LF/HF (b) and parasympathetic nervous system activity, HF (c) were standardized, and mean ± SE of 11 men are shown. For comparison, time course of RQ was shown as red dotted line. Data were derived from a sedentary control trial of a previous experiment focused on the effect of exercise on peripheral clock gene expression (24.5 ± 2.8 years; BMI 22.2 ± 1.9 kg/m^2^; body fat 16.1 ± 3.8%).**^22^** Prescribed diet was designed to achieve an individual energy balance over the 24 h of indirect calorimetry, and provided as breakfast (9:00), lunch (13:00), and dinner (18:00). Subjects slept for 7 h from 23:00 to 6:00 (■).

Appendix Figure 3. 24 h profile of time course of heart rate and autonomic nervous system function in metabolically flexible and inflexible subjects.

Forty-one young men were grouped as metabolically flexible (n=20) or inflexible (n=21) according to the magnitude of range of RQ over the 24 h.**^19-22^** Mean ± SE of heart rate (a), sympathetic nervous system activity, LF/HF (b) and parasympathetic nervous system activity, HF (c) were shown for metabolically flexible (
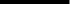
 ) and inflexible (
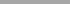
 ) group. Subjects took breakfast (7:00 or 9:00), lunch (12:00, 12:30 or 13:00), and dinner (18:00), and slept for 7 h (23:00-06:00, ■). There were no significant differences in time course of heart rate and its variabilities (LF/HF and HF) between the 2 subgroups.

Appendix Figure 4. 24 h profile of time course of heart rate and autonomic nervous system function in two age groups of 10 years apart.

Fifty-three men were grouped as younger (n=27, under 25 years of age) or older (n=26, 25 years or more).**^9,19-22^** Subjects took breakfast (7:00, 8:00 or 9:00), lunch (12:00, 12:30 or 13:00), and dinner (18:00), and slept for 7 or 8 h from 23:00 (■). Because of unequal duration of sleeping period, the 8^th^ hour of sleep in one experiment was not included for statistical analysis.**^9^** Mean ± SE of heart rate (a), sympathetic nervous system activity, LF/HF (b) and parasympathetic nervous system activity (c) were shown for younger (
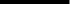
 ) and older (
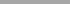
 ) group. There was no statistically significant difference between the two age groups.

Appendix Figure 5. 24 h profile of time course of heart rate and autonomic nervous system function in men and women.

Mean ± SE of heart rate (a), sympathetic nervous system activity, LF/HF (b), parasympathetic nervous system activity and HF (c) in 10 men (
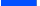
)**^19^** and 9 women (
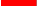
)**^18^** were calculated from a sedentary trial in previous experiments focused on the effect of exercise on 24-h fat oxidation. Since there were large individual variations in HF, mean ± SE of individually standardized HF was also shown (d). Prescribed diet was provided as breakfast (8:00), lunch (12:00), and dinner (18:00), and subjects slept for 7 h from 23:00 to 6:00 (■). There were no significant differences in heart rate and its variability (LF/HF, HF and standardized HF) between men and women.
